# Targeted nanopore sequencing enables complete characterisation of structural deletions initially identified using exon‐based short‐read sequencing strategies

**DOI:** 10.1002/mgg3.2164

**Published:** 2023-03-19

**Authors:** Benjamin McClinton, Laura A. Crinnion, Martin McKibbin, Rajarshi Mukherjee, James A. Poulter, Claire E. L. Smith, Manir Ali, Christopher M. Watson, Chris F. Inglehearn, Carmel Toomes

**Affiliations:** ^1^ Leeds Institute of Medical Research, School of Medicine University of Leeds Leeds UK; ^2^ North East and Yorkshire Genomic Laboratory Hub, Central Lab St James's University Hospital Leeds UK; ^3^ Department of Ophthalmology St James's University Hospital Leeds UK

**Keywords:** *CNGA1*, *CNGB1*, *EYS*, inherited retinal disease, Nanopore sequencing, *PRPF31*

## Abstract

**Background:**

The widespread adoption of exome sequencing has greatly increased the rate of genetic diagnosis for inherited conditions. However, the detection and validation of large deletions remains challenging. While numerous bioinformatics approaches have been developed to detect deletions from whole ‐ exome sequencing and targeted panels, further work is typically required to define the physical breakpoints or integration sites. Accurate characterisation requires either expensive follow ‐ up whole ‐ genome sequencing or the time ‐ consuming, laborious process of PCR walking, both of which are challenging when dealing with the repeat sequences which frequently intersect deletion breakpoints. The aim of this study was to develop a cost‐effective, long‐range sequencing method to characterise deletions.

**Methods:**

Genomic DNA was amplified with primers spanning the deletion using long‐range PCR and the products purified. Sequencing was performed on MinION flongle flowcells. The resulting fast5 files were basecalled using Guppy, trimmed using Porechop and aligned using Minimap2. Filtering was performed using NanoFilt. Nanopore sequencing results were verified by Sanger sequencing.

**Results:**

Four cases with deletions detected following comparative read‐depth analysis of targeted short‐read sequencing were analysed. Nanopore sequencing defined breakpoints at the molecular level in all cases including homozygous breakpoints in *EYS*, *CNGA1* and *CNGB1* and a heterozygous deletion in *PRPF31*. All breakpoints were verified by Sanger sequencing.

**Conclusions:**

In this study, a quick, accurate and cost ‐ effective method is described to characterise deletions identified from exome, and similar data, using nanopore sequencing.

## Graphical abstract

Deletions that are identified by targeted next‐generation sequencing typically need to be characterised to define the physical breakpoints of the deletion. This is vital for the design of a Sanger sequencing assay to validate the deletion; however, this is typically a laborious process. In this study, a quick and cost‐effective method to define a series of deletions using long‐range PCR and nanopore sequencing is described.

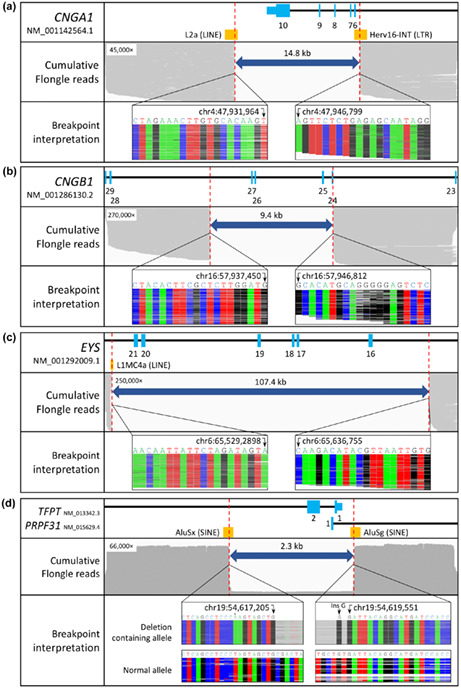

## 1 INTRODUCTION

The ubiquitous adoption of next‐generation sequencing (NGS) has revolutionised the study of genetic disorders. Molecular diagnostic screening is now routinely performed by targeted‐, whole‐exome (WES), or whole‐genome sequencing (WGS), using short‐read instruments predominantly manufactured by Illumina (Chang et al., 2020; Crowley et al., 2020; Kong et al., 2019; Xiao et al., 2021). While WGS is generally regarded as the most comprehensive testing strategy, a targeted focussed approach is typically first utilised before more expensive analyses are deployed. This potentially offers a significant saving on both WGS reagent costs and associated indirect costs, such as data storage and analysis.

For example, an individual with inherited retinal disease (IRD) is often first screened using a disease‐specific capture reagent (Watson et al., 2014) or a “virtual” gene panel from the WES data set. For IRD, there are a range of targeted resources that have proved highly successful in reducing the cost of diagnosing many cases, including the NGS 176 panel (Ellingford et al., 2016; Sheck et al., 2021), the Target 5000 panel (Stephenson et al., 2021) and multiplex amplification strategies like Molecular Inversion Probes (MIPs) (Hardenbol et al., 2003; Hiatt et al., 2013; Reurink et al., 2021; Rowe et al., 2013; Tracewska et al., 2019; Weisschuh et al., 2018). Only the remaining unsolved cases are screened by WGS, greatly reducing the overall time and cost of screening large numbers of patients (Dockery et al., 2021; Haer‐Wigman et al., 2017; Tracewska et al., 2019).

Defining copy number variations (CNVs) from targeted sequencing data remains challenging. Nevertheless, a number of tools that use either a comparative read‐depth or paired end mapping (PEM) approach have been successfully developed (Zhang et al., 2019). In this regard, ExomeDepth allows the number of reads at a locus to be compared to the relative number of reads generated in the other samples sequenced during the same sequencing run (Plagnol et al., 2012). Conversely in PEM, NGS analysis is performed using paired reads that have an expected insert size. Following alignment, if the read's mate creates an unexpectedly large or small interval, is inappropriately oriented, or remains unmapped, a CNV may be suspected (Korbel et al., 2007). Unfortunately, it is common for the boundaries of a CNV to remain uncaptured following hybridisation capture enrichment, but accurate characterisation at nucleotide level is required to confirm and describe the CNV. This also aids Sanger sequencing‐based cascade screening in additional family members.

Traditional methods for defining variant breakpoints include PCR walking; numerous standard PCRs are attempted in the region of the deletion with the aim of spanning the deletion. However, this is time‐consuming, laborious and not always successful. Alternatively, WGS can be used to define the CNV breakpoints, but this remains expensive and is frequently hindered by an inability to unambiguously map short‐read sequences at the target locus, which are often surrounded by low‐complexity sequences (Lauer & Gresham, 2019). The adoption of long‐read sequencing approaches may overcome this limitation (Sun et al., 2020).

Currently, the long‐read sequencing market is dominated by both Pacific Biosciences single molecule real time (SMRT) sequencers and the Oxford Nanopore instruments. Both nanopore and SMRT sequencing are based on single‐molecule sequencing (Ashton et al., 2015; Eid et al., 2009). While PacBio sequencing has a reportedly higher per‐base accuracy (>99.9% single molecule accuracy), the sequencers remain expensive and their running costs are high. By contrast, the MinION, a physically smaller nanopore sequencing instrument, has a considerably lower access cost ($1000). In recent years, subsequent iterations of the pore protein and advances in base calling algorithms have significantly increased the per‐base accuracy from approximately ~64% to 85%–94% (Wang et al., 2021). Furthermore, the introduction of the Flongle flowcell, an adaptor for the MinION sequencing instrument, offers a lower throughput at greatly reduced unit cost, enabling smaller sequencing projects to be undertaken without the need for per‐sample indexing. Although the accuracy of nanopore continues to increase, it remains below that of the sequencing‐by‐synthesis Illumina chemistry.

Here we describe a method to quickly, cost effectively and accurately validate large deletions at nucleotide resolution using long‐range PCR target enrichment combined with nanopore sequencing on a Flongle flowcell. The method was used to validate four deletion‐containing variants detected following comparative read‐depth analysis of targeted short‐read sequencing.

## 2 MATERIALS AND METHODS

### 2.1 Patient recruitment

All patients were diagnosed and recruited to the study by ophthalmologists at St James's University Hospital, Leeds, UK. Blood samples were collected from patients and family members after obtaining informed consent. Ethical approval was provided by the Leeds East Teaching Hospitals NHS Trust Research Ethics Committee (Project number 17/YH/0032). Genomic DNA was isolated by Yorkshire Regional Genetics using standard protocols.

### 2.2 Nanopore breakpoint identification

Standard PCR primers were designed using Primer3 (http://primer3.ut.ee/) and validated using the UCSC in silico PCR tool (https://genome.ucsc.edu/cgi‐bin/hgPcr). All primers were purchased from Sigma‐Aldrich with desalted purification. Primer sequences are recorded in Supporting Information Table S1.

Long‐range PCR was performed using the SequalPrep™ long PCR Kit (Thermo‐Fisher) following the manufacturer's guidelines. All long‐range PCRs were performed using 35 rounds of thermocycling. An Agencourt AMPure XP bead clean‐up (Beckman Coulter™) was performed to remove unligated adapters and unamplified DNA fragments.

Sequencing libraries were prepared using the SQK‐LSK109 ligation sequencing kit (Oxford Nanopore Technologies [ONT]). Bead‐based washes were performed using Long Fragment Buffer and the final library was eluted in 6 μL of Elution Buffer, following a 10‐min incubation at room temperature. ONT sequencing was performed using a Flongle Flowcell (R.9.4.1) and MinION instrument.

Basecalling was performed using Guppy v.5.0.16 (https://nanoporetech.com/). Adaptor sequences were removed using Porechop v.0.2.4 (https://github.com/rrwick/Porechop). Read processing was performed using NanoFilt v.2.8.0 (De Coster et al., 2018); the first 75 bases of each read was trimmed and those reads shorter than 2000‐bp and longer than 12,000‐bp were removed. Reads with a quality score less than Q10 were also filtered. Sequencing metrics were generated using NanoStat v.1.5.0. Alignment‐ready reads were mapped to the human reference genome (build hg19) using MiniMap2 v.2.22 (Li, 2018). Files were manipulated using Samtools v.1.9 (Li et al., 2009), and aligned reads were visualised using the Integrated Genome Viewer v.2.7.2 (Robinson et al., 2011). BLAT (invoked through IGV) was used to obtain primer co‐ordinates and visualise sequence context. Repetitive elements were identified using UCSC Genome Browser (http://genome.ucsc.edu).

### 2.3 Sanger sequencing verification

For all cases, a PCR was designed to span the deletion breakpoint. PCR reactions were performed in a 25 μL final volume. This comprised 10 μmol of forward and reverse primer, 200 μM of each dNTP, 1× PCR reaction buffer (Invitrogen), 1.0 mM or 1.5 mM MgCl_2_ (Invitrogen), 1 unit of Taq Polymerase (Invitrogen) and 50 ng of genomic DNA made up to 25 μL with nuclease‐free water. Thermocycling conditions consisted of a denaturation step at 96°C for 3 min followed by 30–35 amplification cycles comprising 92°C for 30 s, 60–65°C for 30 seconds and 72°C for 30 s before a final extension step at 72°C for 10 min. For all cases, the long‐range PCR amplicon was directly sequenced using internal sequencing primers.

Sanger sequencing reactions were performed using BigDye Terminator v.3.1 and resolved on an ABI3130xl Genetic Analyser (Applied Biosytems) according to the manufacturer's instructions. Electropherograms were visualised using 4Peaks (https://nucleobytes.com/4peaks/).

## 3 RESULTS

We sought to develop a simple and effective workflow to enable quick and accurate characterisation of heterozygous and homozygous deletion variants, using IRD patients as our exemplar cohort. Cases 1–3 were initially sequenced using a MIPs panel targeting 100 IRD genes (Weisschuh et al., 2018). Case 4 was initially sequenced by WES. All of the deletions were identified using ExomeDepth, which compares normalised read depths from multiple exomes, to detect CNVs. A number of primer pairs were designed for each case. Primers were designed to flank the putative deleted regions by positioning them within the exonic sequence captured on either side of the deleted exons and randomly within the intronic sequence flanking the deleted exons. For all cases, at least one primer pair resulted in an amplified product smaller to that expected from the reference sequence, indicating the deletion breakpoints had been amplified.

**FIGURE 1:**
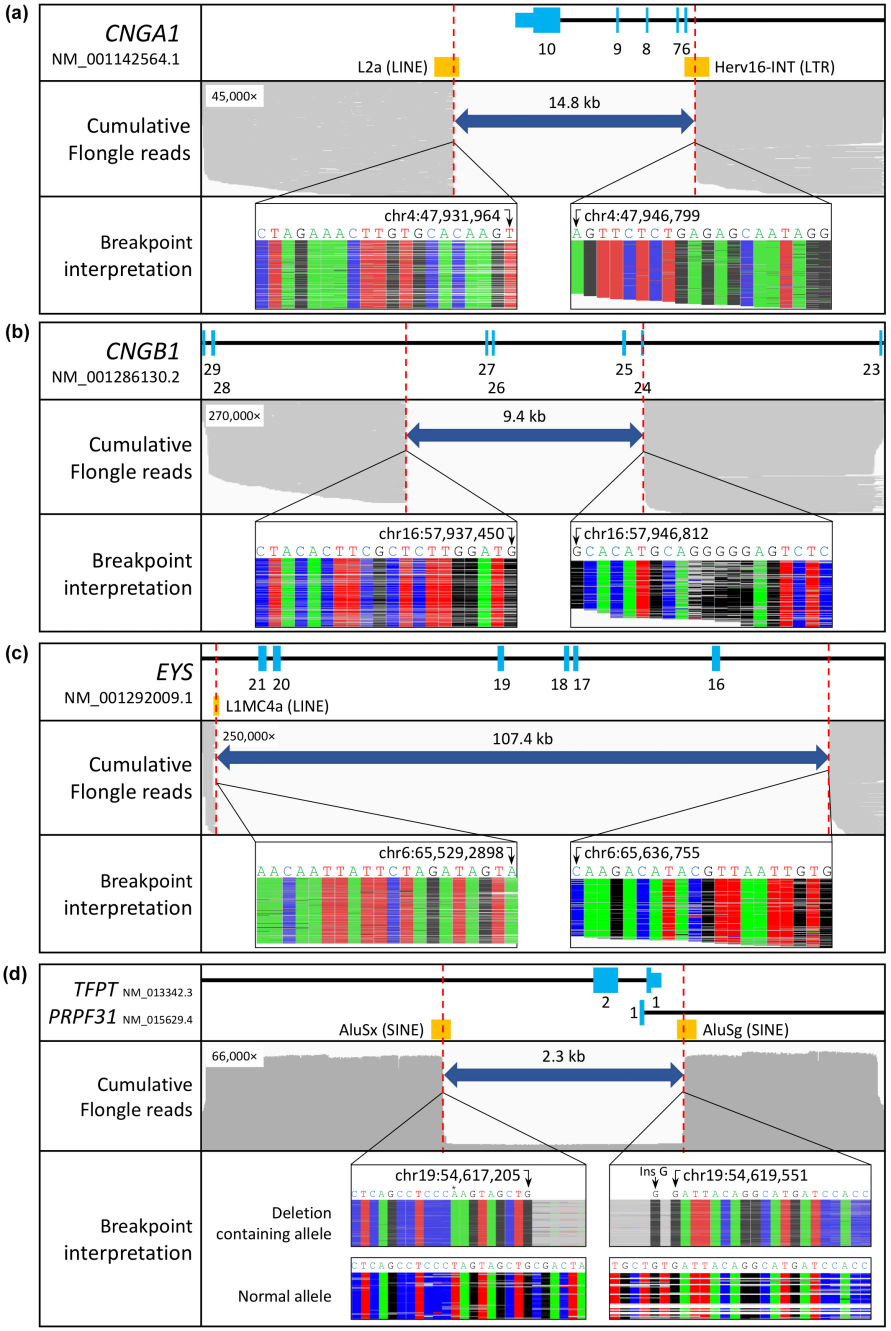
(a) Characterised deletion in *CNGA1*. (b) Characterised deletion in *CNGB1*. (c) Characterised deletion in *EYS*. (d) Characterised deletion in *PRPF31*. The top track of each panel shows a schematic representation of the deletion at the target locus. The middle panel shows the reads as viewed in IGV. Grey bars represent the sequenced reads, except in panel D, where they represent the cumulative read depth. For each case, the *y*‐axis cumulative read depth is as indicated in the panel. The reads shown are a 1% down‐sample of the total reads sequenced. The bottom panel shows the nanopore sequence at the breakpoint. For the heterozygous case in panel d, variant‐containing and normal reads have been separated. A point mutation on the same allele is indicated by a *.

Case 1 was a single case of retinitis pigmentosa. Initial MIPs sequencing indicated a homozygous deletion spanning exons 6–10 of *CNGA1*. The minimum size of the deletion was estimated to be 7.5 kb, and the maximum size of the deletion was estimated at 56 kb. The deletion was confirmed by long‐range PCR across the locus which yielded an estimated 9 kb product instead of the 25 kb expected for the reference allele. Nanopore sequencing of the long‐range PCR product enabled characterisation of the breakpoint at nucleotide resolution (Figure 1a), defining a novel 14 kb homozygous deletion that included exons 6–10 of *CNGA1*, (NM_001142564.1) g.47931965_47946798del (hg19). More than 71% of the encoded amino acids in the indicated transcript were deleted, likely representing a total loss of function. A maximum read depth of 44,936 and a mean read depth of 23,810 was achieved across the locus (Table 1).

**TABLE 1:** Summary sequencing metrics for nanopore sequencing runs.

Case	Target gene	Reads generated (MinKNOW) (K)	Median Q score^a^	Read length N50	Proportion of reads on target (%)	Maximum read depth (×)	Mean read depth at target locus (×)
Case 1	*CNGA1*	269.91	11.4	9458	0.735	44,936	23,810
Case 2	*CNGB1*	325.54	12.7	4432	0.998	273,742	202,575
Case 3	*EYS*	327.21	13.8	4203	0.999	250,203	224,065
Case 4	*PRPF31*	378.39	14.1	5799	0.622	66,304	44,365

^a^ Of reads following length and quality filtering. Filtering on a quality score greater than 10 was used.

Case 2 had a diagnosis of autosomal recessive retinitis pigmentosa. MIPs sequencing indicated a homozygous deletion of exons 25–27 of *CNGB1*. The estimated minimum size of the deletion was 8 kb and the maximum size was 11 kb. Long‐range PCR yielded an approximately 5 kb product (smaller than the 14 kb reference allele) and nanopore sequencing characterised a novel 9.4 kb deletion encompassing exons 25–27 of *CNGB1*, (NM_001286130.2) g.57937451_57946811del (hg19) (Figure 1b). This is an out of frame deletion in *CNGB1*, again likely indicating a loss of function. A maximum read depth of 273,742 and a mean read depth of 202,575 was achieved across the locus (Table 1).

Case 3 had a diagnosis of sporadic retinitis pigmentosa. Initial MIPs sequencing indicated a homozygous deletion of exons 16–21 of *EYS*. The minimum size of the deletion was estimated to be 91 kb, and the maximum size of the deletion was estimated to be 137 kb. Long‐range PCR generated a 5 kb product (smaller than the 112 kb reference allele). Nanopore sequencing characterised a novel 107 kb deletion encompassing exons 16 to 21of *EYS*, (NM_001292009.1) g.65529289_65636754del (hg19) (Figure 1c). This deletion is likely to be deleterious as it overlaps the calcium‐binding domain of the protein (https://www.uniprot.org/uniprot/Q5T1H1#family_and_domains, accessed 9/6/2022). A maximum read depth of 250,303 and a mean read depth of 224,065 was achieved across the locus (Table 1).

Case 4 had a diagnosis of autosomal dominant retinitis pigmentosa. WES identified a putative heterozygous deletion of the first non‐coding exon of *PRPF31*. The deletion was estimated to be a maximum of 8.6 kb based on available sequence data. The deletion was confirmed by long‐range PCR across the suspected locus; two bands were visible in the affected heterozygous cases versus one band in the control. Nanopore sequencing of the PCR products characterised a 2.4 kb heterozygous deletion encompassing exon 1 of *PRPF31* as well as exons 2 and 3 of the neighbouring gene *TFPT*, (NM_015629.4) g.54617206_54619550delinsG (hg19) (Figure 1d). A maximum read depth of 66,304 and a mean read depth of 44,365 was achieved across the locus (Table 1). The deletion appears to be novel although pathogenic deletions encompassing the first non‐coding exon of *PRPF31* have recently been reported, but incompletely characterised (Ruberto et al., 2021). After characterising the deletion using nanopore sequencing, a long‐range PCR assay was used to segregate the deletion in a pedigree (Supporting Information Figure S2).

For all cases, Sanger sequencing was used to validate the long‐read sequencing data and confirm the breakpoints (Supporting Information Figure S1). All four breakpoints either overlapped or were located close to low‐complexity repeats.

## 4 DISCUSSION

WES, and similar targeted hybridisation enrichment strategies, have revolutionised diagnostic sequencing strategies. Further advances continue to be made, both in short‐read sequencing and subsequent analysis, but diagnostic challenges remain. One of these is the characterisation, at nucleotide resolution, of incompletely resolved structural variants, without resorting to expensive and time‐consuming follow‐up experiments like WGS. In this study, we have used long‐range PCR and Flongle‐based nanopore sequencing to develop a simple and low‐cost method that can be deployed to characterise the breakpoints of deletion variants identified using ExomeDepth or similar algorithms.

We characterised three homozygous deletion variants and a heterozygous deletion variant initially identified from either WES or MIPs data sets. ExomeDepth analysis identified the presence of deletions of between 7.5 and 137 kb, but as the exact breakpoints were located in introns they were not captured. In all cases, we were able to PCR the surrounding locus by positioning amplification primers in the flanking sequence. Amplification products were then nanopore sequenced.

Nanopore sequencing has an increased error rate compared to short‐read synthesis‐by‐sequencing NGS chemistry (Wang et al., 2021). However, the per base accuracy of nanopore sequencing has significantly increased rapidly in recent years. Additionally, the considerable depth‐of‐coverage achieved from the sequencing runs (40,000× − 270,000×) meant that consensus sequences could be straightforwardly determined; the breakpoints were also verified by Sanger sequencing. As this was a pilot study, only one sample per Flongle was used. However, the excess capacity could be used to pool multiple samples; presently only 1% of the available reads at the locus were analysed and visualised. Library indexing may not be required if different genes are being characterised, as was the case in this study. The utilisation of the Flongle, which offers a lower output at a fraction of the cost of a MinION flowcell, makes this a relatively inexpensive method to accurately define the breakpoints of deletion‐containing variants. At a per Flongle cost of £56 (plus library preparation reagents of ~£40 per sample), this is an extremely cost‐effective method even without multiplexing.

Alternative long‐read methods such as low coverage WGS have been demonstrated to be effective at characterising structural variants, but these methods remain comparatively expensive (Lavrichenko et al., 2021). A number of amplification free enrichment methods are currently being used in conjunction with Oxford Nanopore sequencing to enable sequencing of native genomic DNA. These methods include CRISPR‐Cas9‐mediated enrichment (Gilpatrick et al., 2020; Watson et al., 2019), CATCH (Gabrieli et al., 2018; Jiang et al., 2015) and ReadFish adaptive sampling (Payne et al., 2021). While these methods are powerful and allow for long‐read sequencing while reducing issues surrounding the introduction of PCR artefacts and PCR length restrictions, they are all currently performed on a Nanopore MinION flowcell to ensure sufficient depth of coverage. The MinION flowcell is significantly more expensive than the Flongle flowcell, making this technique prohibitively expensive for routine use. In addition, these techniques require a large amount of DNA which often has to be extracted using specialist methods (Gong et al., 2019) and is therefore not available for all samples.

Low‐complexity repeat sequences are a major driver for the creation of CNVs. Indeed, the presence of Alu repeats at the breakpoints in case 4 (*PRPF31*) indicates that non‐allelic homologous recombination may have caused the genomic rearrangement in this case (Peng et al., 2015). Similarly, the other three deletions all either overlapped a repetitive element on at least one side of the breakpoint or were situated very close to repetitive elements. This has been hypothesised to lead to the presence of secondary structures, causing replication fork stalling or collapse, thereby leading to the formation of deletions (Khan et al., 2020; Vissers et al., 2009). A major advantage of the breakpoint characterisation method described here is that it circumvents issues caused by the presence of repetitive elements at the breakpoints of deletions, which can cause issues aligning short‐read WGS data, generating PCR products, and subsequently Sanger sequencing across amplicons with internal primers (Lavrichenko et al., 2021). Long‐read sequencing overcomes the issue of sequencing difficult repeats and provides more leeway to avoid repetitive elements when designing primers.

A limitation of the approach is the need to PCR amplify across the CNV, given the size limit to this technology and the preferential amplification of smaller products. This approach works well for deletion variants because smaller amplification products resulting from the presence of the deletion will preferentially amplify compared to the wild‐type product. Case 4 with the *PRPF31* heterozygous deletion demonstrates this, as there is over 10× more coverage of the allele with the deletion than the wild‐type allele with no deletion present (66,304× and 6150× maximum, respectively). The corollary of this is that insertions will be difficult to characterise because the wild‐type product will preferentially amplify. This will therefore make the characterisation of larger insertions challenging.

In summary, we have demonstrated that long‐range PCR and Flongle‐based nanopore sequencing is a simple, quick and efficient method to characterise CNVs, especially deletions, identified from exon‐based NGS screening strategies.

## AUTHOR CONTRIBUTIONS

BMC performed study concept and design, performed experiments, analysis and writing of the paper, CMW performed study concept and design, development of analysis and, review and revision of the paper, LAC provided technical support, performed experiments and provided analysis of the data, MMK and RM provided patient samples and clinical information and analysis of the data, JAP and CELS performed experiments and analysis of the data, CT, MA and CFI obtained funding, performed study concept and design, provided analysis and interpretation of data and, review and revision of paper. All authors read and approved the final paper.

## ACKNOWLEDGMENT

We thank the families for their participation in this study. This work has been supported by a Horizon 2020, Marie Sklodowska‐Curie Innovative Training Network entitled European Training Network to Diagnose, Understand and Treat Stargardt Disease, a Frequent Inherited Blinding Disorder‐StarT (813490). Christopher Watson is supported by the NVIDIA Academic Hardware Grant Program.

## CONFLICT OF INTEREST STATEMENT

Dr Watson has received travel expenses to speak at an Oxford Nanopore Technologies organised conference. The authors have no other financial/conflicting interests to disclose.

## ETHICS STATEMENT

Ethical approval was provided by the Leeds East Teaching Hospitals NHS Trust Research Ethics Committee (Project number 17/YH/0032) and followed the tenets of the Declaration of Helsinki. Written informed consent was obtained from the participants.

## Supporting information


Figure S1.

Table S1.

## Data Availability

The data that support the findings of this study are available from the corresponding author upon request.
