# Supplementary material for: Targeted nanopore sequencing enables complete characterisation of structural deletions initially identified using exon‐based short‐read sequencing strategies
Source: Mol Genet Genomic Med. 2023 Mar 19;11(6):e2164. doi: 10.1002/mgg3.2164 (PMC10265035; doi:10.1002/mgg3.2164)
Supplement: Supplementary file 1 — Figure S1. [file MGG3-11-e2164-s002.docx]

**Supplementary Figure 1: Sanger sequencing verification of deletion breakpoints. (A)** Electropherogram at breakpoint in homozygous *CNGA1* deletion. **(B)** Electropherogram at breakpoint in homozygous *CNGB1* deletion **(C)** Electropherogram at breakpoint in homozygous *EYS* deletion. **(D)** Electropherogram at breakpoint in heterozygous *PRPF31* deletion. In each panel, the Sanger sequencing chromatograms in the top row show the sequence at the breakpoint in a control case, with the location of the deletion breakpoint indicated by a dashed line and the adjacent genomic coordinates. The trace in the bottom row in each panel shows the same locus in the case with the deletion. In case 4, a T>A variant at chr19:54,617,197 is circled. Additionally, a one base insertion of a G nucleotide is indicated by the “*”. Genomic coordinates are provided according to the human reference genome (build hg19).

**Supplementary Figure 2: Segregation of *PRPF31* deletion using long range PCR:** A) Electrophoresis gel showing the segregation of the deletion in *PRPF31* using long range PCR. B) Family pedigree of case 4 (indicated by arrow, not shown on gel), showing segregation of the mutation with ophthalmologist confirmed disease status. Previously reported deletions in the first non-coding exon of *PRPF31* show reduced penetrance. 5360 carries the deletion but did not appear affected at time of examination. The rare familial second name of the proband prompted investigation of a subsequently ascertained family. Confirmation of the PRPF31 deletion in individual 4395 suggests distant relatedness (as denoted by the dashed line).
